# Research Advances in the Application of AI for Preoperative Measurements in Total Knee Arthroplasty

**DOI:** 10.3390/life13020451

**Published:** 2023-02-06

**Authors:** Wei Li, Sheng-Ming Xu, De-Bao Zhang, Huang-Yi Bi, Gui-Shan Gu

**Affiliations:** Department of Orthopaedics, The First Bethune Hospital of Jilin University, Street Xinmin 71, Changchun 130021, China

**Keywords:** knee osteoarthritis, total knee arthroplasty, AI measurement

## Abstract

Total knee arthroplasty (TKA) is widely used in clinical practice as an effective treatment for end-stage knee joint lesions. It can effectively correct joint deformities, relieve painful symptoms, and improve joint function. The reconstruction of lower extremity joint lines and soft tissue balance are important factors related to the durability of the implant; therefore, it is especially important to measure the joint lines and associated angles before TKA. In this article, we review the technological progress in the preoperative measurement of TKA.

## 1. Introduction

Total knee arthroplasty (TKA) is the most conclusive treatment for severe knee osteoarthritis, and its efficacy relies on the reconstruction of joint lines and soft tissue balance in the lower extremity. The reconstruction of the knee joint, among various structures in the human skeletal and soft tissue systems, is the most complex, for it requires the integration of anatomy, kinesiology, biomechanics, and even tissue engineering. In the reconstructed knee, it is important to take into account the reconstruction of joint lines, the soft tissue balance, and patellar tracking so as to achieve the “forgotten knee” status. The ultimate goal of accurate reconstruction is pursued by orthopaedic surgeons of all ages. 

In order to achieve more accurate alignment, more perfect soft tissue balance, and better prosthesis matching so as to obtain longer prosthesis life, better postoperative function, and higher patient satisfaction, AI-assisted TKA preoperative design can accurately carry out preoperative planning, artificial intelligence segmentation, and key point recognition of joint CT data and establish standardized measurement model by adjusting the joint orientation. After introducing the open/closed wedge parameters or prosthesis parameters, one must accurately judge the shape parameters, such as the height and inclination of the osteotomy surface, and finally form a highly reliable surgical prediction model and simulate the postoperative image.

Therefore, thorough preoperative planning, that is, the measurement of joint lines and angles, helps guide the surgeon during the procedure. Preoperative measurements taken for TKA include traditional plain film radiographic measurements, digital templating, computer navigation, and artificial intelligence (AI) measurements. AI has multiple definitions and is largely agreed upon as the intelligence demonstrated by machines. In the field of computer science, AI research is defined as the study of “intelligent agents”, which are “devices that perceive the environment and act to maximize the achievement of a goal”. In a broad sense, process digitization, computer navigation, and robotics all fall into the category of AI. Existing AI-assisted technologies have enabled modern medicine to make great strides toward precision, intelligence, and individualization. 

This article reviews the advances in AI-based preoperative measurement for TKA and the problems associated with various measurement techniques.

## 2. Traditional Plain Film Radiographic Measurements

In the past, preoperative measurements for TKA relied on traditional plain film radiographic measurements. Preoperatively, a full-length film of the patient’s bilateral lower extremities was taken in the standing position along with the anteroposterior (AP) and lateral views of the affected knee joint and an axial film of the patella. Further, the surgeons needed to manually trace the femoral anatomical axis, tibial axis, and lower limb joint lines on the full-length film of bilateral lower limbs to determine the valgus angle. The disadvantage of this type of film template measurement based on X-ray films is that the magnification of X-ray images varies among different hospitals, thus affecting the accuracy of measurements made using the same film template. The basis for testing the practical value of preoperative template measurements is that the results of different surveyors are consistent under different conditions [1,2]. Studies have shown that the inter- and intra-rater variability at different times are as high as 46.8% and 43.6%, respectively; thus, for the initial TKA, the accuracy and reproducibility of preoperative template measurements are not high [3]. Moreover, the quality of the preoperative imaging film also affects the accuracy of the measurements. Traditional film templates have a fixed magnification of 115–120%, which often does not match the actual radiographic magnification. In addition, due to differences in the body type and posture of the patients, it is difficult to ensure that the radiographs are obtained at a constant magnification and match the film template, especially in patients with severe knee osteoarthritis and flexion contracture. Moreover, if the patient has an extra-articular deformity on the femoral or tibial side, the only position for AP projection of both lower extremities is with the patella facing forward, which can result in human errors in determining the anatomical axes of the femur and tibia.

## 3. Digital Templating Measurements

The initial digital measurement is based on the measurement function that comes with each hospital’s medical imaging information system. These measurements require full-length films of both lower extremities, AP, and lateral knee films. Instead of traditional film measurements, computed tomography (CT) images of lower limbs need to be taken. The joint line and valgus angle of the lower extremity are measured in the same way as in conventional film measurements. The difference is that the medical imaging information system allows accurate labelling of the trans-epicondylar axis (TEA) and the Whiteside’s line based on CT images of the lower extremity at the selected knee level, and the external rotation angle in osteotomy can be determined by the posterior condylar line (PCL) of the knee. However, in the past, based on the preoperative CT markings of the TEA and the Whiteside’s line or the default 3° of external rotation for osteotomy, the intraoperative accuracy was 90%, 83%, and 70%, respectively. 

The medical imaging information system allows only preoperative measurements of all angles but does not simulate the intraoperative knee osteotomy and the post-osteotomy prosthesis placement. In TKA, according to the isometric osteotomy method, the specific thickness of the osteotomy also affects the choice of the size of the prosthesis. The accuracy of the digital planning software in predicting the size of tibial prosthesis has been reported to be as low as 63%, and that in predicting the size of femoral prosthesis is 69% [4]. Schotanus et al. [5] similarly concluded that the outcome of digital preoperative planning was yet to be determined. 

In the field of orthopaedic surgery, digital technology has made it possible to digitally simulate surgery. The CT scan of the patient’s knee joint, with a slice thickness of 1 mm, is obtained in the DICOM format, and the obtained data are input into the corresponding digital software to reconstruct a three-dimensional (3D) bony model of the knee joint. Further, calibrated weight-bearing full-length AP and lateral radiographs of the lower limb are taken, and the statistical shape model (SSM) technique is used to derive the 3D model of the knee joint. The 3D model is then automatically aligned with the CT-reconstructed bony 3D model of the knee joint to obtain a 3D spatial model of the knee joint and the full-length of bilateral lower limbs. After scanning all models of the selected prosthesis, the installation and alignment of the prosthesis are simulated on the 3D model using the measured joint lines and angles. The application of such digital simulation technology in the preoperative measurement of TKA has enabled the lower extremity joint line measurement and osteotomy and prosthesis installation to be more accurate, which is a great leap forward in joint surgery measurement technology. It has been confirmed [6,7] that the deviation of the SSM technique in fitting the knee joint is less than 0.2–0.4 mm, which can meet the rigorous standards required for clinical application.

## 4. Computer Navigation Technology-Aided Measurement

In computer-assisted orthopaedic surgery, information contained in the multimodal imaging data is digitally processed and combined with stereo-navigation systems to identify and display anatomical structures. The computer plans the surgical path and formulates a surgical plan for preoperative surgical simulation under the guidance of appropriate image monitoring and stereo-navigation systems. A certain guidance system is used to perform intraoperative surgical interventions, wherein computers and medical robots assist surgeons to complete the operation. Numerous studies have confirmed that computer navigation-assisted total knee arthroplasty (TKA) can improve the accuracy of lower extremity alignment and prosthesis placement [8,9,10,11].

In the field of orthopaedics, computer-aided systems were first used in spine surgery, and Saurol et al. first developed and applied a pedicle screw navigation system in 1992. In 1993, research on knee surgical navigation systems began in France, and Sal’agaglia’s [12] group pioneered the development of a knee surgical navigation system that did not require imaging data. Consequently, in 1997, the first computer-assisted TKA was performed successfully. The earliest computer navigation systems were based on CT images and were used in clinical surgery. 

Computer navigation systems can be divided into two broad categories: The first is based on imaging data (CT and MRI), wherein preoperative imaging is entered into the computer, and preoperative images based on the recorded anatomical landmarks are compared with the intraoperative morphology. The second type of navigation system does not require imaging data. Instead, it contains a large amount of anatomical data and determines the movable surface of the knee and the alteration of the biomechanical axis of the femur and tibia through passive flexion and extension activities of the knee joint [13]. The difference between the two in actual surgery is that the former facilitates more precise preoperative planning, while the latter facilitates intraoperative joint line control, soft tissue balance, and restoration of joint mobility [14].

The main knee joint navigation systems currently used in China include OrthoPilot^®^, an image-free, infrared-based computer navigation system from Aesculap (Mersongen, Germany), VectorVision^®^ from Brainlab, and the navigation system from Stryker (Klamatsu, MI, USA). Computer navigation systems can accurately calculate the patient’s hip–knee–ankle angle and clearly mark the lower extremity joint lines and the mechanical axes of the femur and tibia, ensuring accurate osteotomy intraoperatively. As per a meta-analysis by Mason et al. [9] including 29 studies with 61 treatment groups and 3437 patients, computer-navigated TKA significantly improved the lower extremity mechanical axis and position of the prosthesis compared with conventional surgery: 9.0% of patients in the navigation group had a postoperative lower extremity mechanical axis malalignment greater than 3° compared with 31.8% in the conventional surgery group. Similarly, femoral and tibial prostheses’ malalignment greater than 3° occurred in 9.6% and 4% of patients in the computer navigation group, respectively, and 11.1% and 34.1% of patients in the conventional surgery group, respectively. Furthermore, the computer navigation system was shown to be advantageous for the reconstruction of the rotational force line and patellar tracking of the lower limb; this was made possible due to real-time navigation and positioning of the affected limb, which helped evaluate the knee motion trajectory and the joint line of the lower limb [15,16].

Although computer navigation systems can improve the accuracy of joint line estimation and prosthesis placement in TKA, there are no clear studies showing that the application of computer navigation significantly improves lower extremity function after TKA. Accelerometer-based portable navigation systems, such as OrthAlign™ and iASSIST^®^, have the advantages of a shorter operation time, shorter learning curve, and lower cost over traditional computer navigation. The perception of spatial position is through precise accelerometers and gyroscopes, and some studies have shown that this system has significant advantages in osteotomy of the femoral condyle and tibial plateau [17,18]. Iorio et al. [19] performed TKA in 53 patients with the assistance of the accelerometer-based navigation system and showed that the tibial component of the prosthesis was aligned within 3° vertically, relative to the mechanical axis, in all patients. Nam et al. [20] also indicated that the portable navigation system was equally accurate in femoral osteotomy. In their study, measurements on 48 patients showed a mean absolute difference of (0.8 ± 0.6)° between the intraoperative goal of 0° and the actual postoperative femoral component alignment measured on radiographs, with 95.8% positioned within 2° of the intraoperative goal and 100% positioned within 3° of the intraoperative goal. However, a limitation of the iASSIST^®^ navigation system is that it is not able to plan the type of prothesis to be used in advance and requires the surgeon to decide on the type of prothesis during the surgery using traditional surgical methods. The system requires intraoperative rotation of the limb to obtain the 13 femoral positioning points for calculating the centroid of the femoral head, which, together with other intraoperative positioning manoeuvres, takes more time than conventional surgery.

Notably, the development of novel computer navigation systems has risen markedly in recent years. The new computer navigation system, Knee 3 software, from Brainlab, Germany, was launched in China in 2020. The system comes with the advantage of simple and fast registration. It enables intraoperative visualization of osteotomy positioning and dynamic real-time display of knee flexion and extension gaps throughout the procedure, which helps the surgeon create individualized lower extremity alignment and soft tissue balance in patients. Compared with conventional computer navigation systems, Knee 3 software not only ensures accurate osteotomy but also improves the soft tissue balance in the knee joint. The Knee 3 system provides real-time intraoperative display of the joint gap in the form of gap values and gap graphs, allowing the operator to visually assess the knee flexion and extension gap balance. When planning the surgery, the surgeon can adjust the angle and position of the femoral and tibial osteotomy according to the patient’s lower extremity joint lines, and the system can also display the impact of the corresponding osteotomy changes on the joint space in real time. The disadvantage of the Knee 3 system is that it includes system setup, tracker fixation, and registration steps. Moreover, since the Knee 3 software dynamically displays the lower limb joints line and knee gap information in real time, the operator needs to quickly interpret the relevant information and devise a suitable surgical plan, which increases the operation time. In addition, because computer navigation-assisted surgery requires the installation of fixation pins to fix the tracker, there is a risk of fracture [21,22,23,24]. Smith et al. [25] reported that during computer navigation system-assisted and robot-assisted TKA, the incidence of fractures related to fixation pins was 0.06%–4.8%, and most of the fractures occurred in the femoral shaft. In recent years, AI deep learning technology has been successfully applied in the field of medical image processing, realizing automatic recognition and segmentation of the lesion or target area, and with high accuracy. AI deep learning technology was applied to independently build neural network PointRend_ Unet on the basis of ensuring the accuracy and robustness of segmentation, which realizes the fast segmentation of knee joint CT image data to improve the work efficiency and reduce the preparation cost. The segmentation results obtained by clinical evaluation are satisfactory, and the steps are as follows: First, establish a CT image database, import a large number of knee joint CT images of patients into Mimics software (Materialise Company-Technologielaan 15, Heverlee, Leuven, Flemish, Belgium), reconstruct the bone structure according to the bone threshold setting on the basis of the threshold method, select the target bone structure, and then conduct threshold growth segmentation; then, conduct manual trimming, and finally, save the data in mask format. Secondly, the neural network is built and trained. Through the segmentation of the neural network, the knee joint CT image data form the femur, tibia, fibula, and patella regions, respectively. Finally, the visualization of the target bone structure 3D model is generated through 3D reconstruction technology. Although non-imaging mode and ultrasound and other non-radiation exposure technologies are emerging, the new TKA auxiliary technology commonly used in clinical practice is based mainly on CT images, which extract the three-dimensional anatomical model of the knee joint from the patient’s CT images for subsequent surgical planning. The manual processing of CT image data is time-consuming and laborious. The realization of simplified, automatic, and accurate segmentation of knee joint CT image is the key to the wide range clinical application of TKA new auxiliary technology. The development of artificial intelligence technology has led to the development and research of a large number of medical image automatic segmentation systems. The commonly used neural networks are deeplab or Unet. These methods have effectively improved the stability of automatic segmentation and the efficiency of the treatment plan workflow.

On 19 July 2022, Enovis announced the launch of ARVIS (augmented reality visualization and information system) and successfully completed more than 200 clinical cases in the United States. This FDA-approved system is the only proprietary real-time hands-free augmented reality (AR) technology designed for surgeons, allowing them to conduct real-time visualization and precise guidance of hip and knee joints with the support of AR. AR technology is a form of surgical guidance technology, which can help surgeons place and align implants during surgery. Unlike other traditional robot systems that require additional personnel, ARVIS is an independent wearable surgical guidance device controlled by surgeons, which can be worn on the ARVIS headband and is compatible with helmets that can be worn during surgery, thus ensuring that surgeons can focus on their patients through the surgical view. ARVIS is the first system with proprietary hardware designed to help surgeons accurately place hip and knee replacements to help improve joint replacement recovery results. ARVIS is more sustainable and environmentally friendly than other technologies because it eliminates disposable plastic instruments and consumables that need to be handled and is approved in the United States for navigation of total hip, total knee, and single-space knee arthroplasty. ARVIS can provide AR navigation support for orthopaedic doctors, such as visual navigation during knee surgery.

## 5. Patient-Specific Instrumentation Measurement Technology

**Figure 1:**
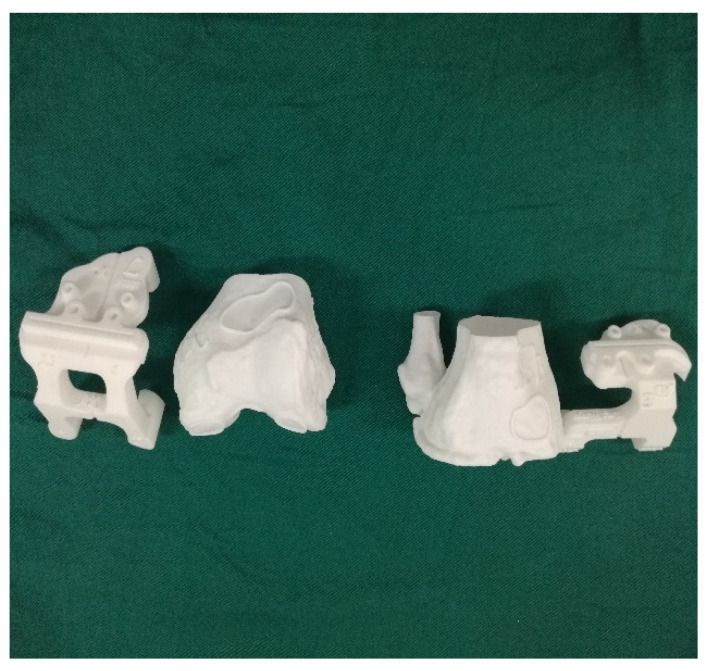
3D printing pre-operative template.

**Figure 2:**
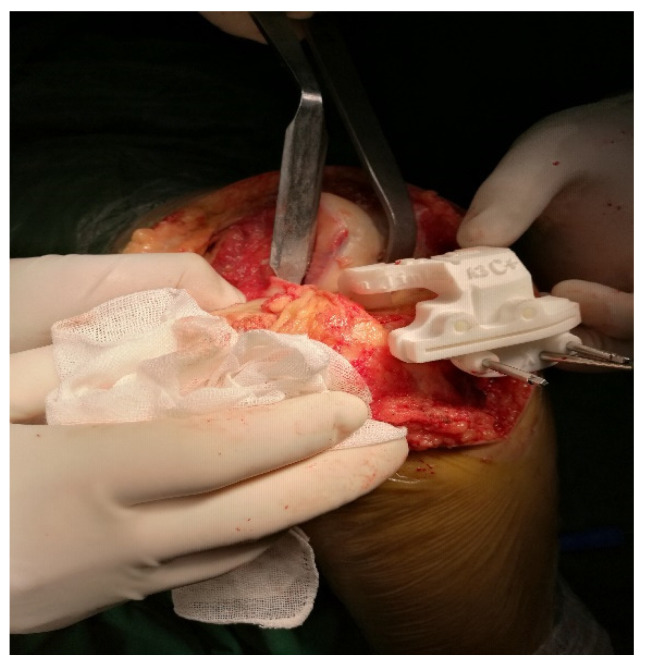
Intraoperative application of 3D printing template.

Some scholars do not support the inclusion of patient-specific instrumentation (PSI) under the category of artificial intelligence, though the authors do not agree with this view. PSI also requires data on the hip joint, knee joint, and ankle joint of the patient to be obtained through CT imaging, which are then digitally processed. The two-dimensional data are reconstructed into a 3D model, and then the corresponding osteotomy volume, angle, and prosthesis type are determined. After planning, the PSI is automatically generated, and the personalized osteotomy surgical guide is obtained via 3D printing technology (Figure 1 and Figure 2). 

Several studies have shown that the use of 3D-printed osteotomy guides can achieve favourable lower limb force lines during TKA [26,27], shorten the operation time, and achieve good results in controlling the femoral component rotation and tibial slope [28,29,30] and pre-operative planning of the required prosthesis type. However, TKA with PSI takes into account only the osteotomy aspect, while the soft tissue release and balance need to be controlled by the surgeons on the basis of their surgical experience. The PSI is based on CT and on bony landmarks. Intraoperatively, however, the guide may be inaccurately attached due to soft tissue coverage or obscuration. The future of this technique involves the inclusion of nuclear magnetic imaging.

## 6. Preoperative Planning of Robot-Assisted TKA 

Before robot-assisted total knee arthroplasty, the AIHIP/KNEE software system was used in the preoperative planning of hip arthroplasty or knee arthroplasty. The system applies computer 3D data analysis based on CT or MRI, applies computer depth learning algorithm to segment joint CT data and identify key points, establishes a standardized measurement model, and then introduces prosthesis parameters to accurately judge parameters, such as osteotomy plane height and posterior inclination angle.

Institutions performing robotic research in China and abroad have explored the introduction of robotic surgery in arthroplasty, and the use of robot-assisted TKA systems is an historic moment. Robot-assisted TKA is currently the highest-level embodiment of artificial intelligence (AI) in knee surgery.

AI technology was once limited by basic hardware, barren databases, deficits of algorithms, and so on.

Alan Turing first invented artificial intelligence in 1950. He established the Turing test, which is the earliest prototype of AI. Turing proposed that the concept of AI technology is a computer algorithm similar to human brain intelligence, but at the same time, its complexity and efficiency are no less than or even higher than human brain intelligence. At that time, due to the backwardness of basic hardware equipment, poor databases, lack of algorithms, and other limitations, the development of AI technology was basically at a standstill. However, in recent years, the rapid development of computer technology and Internet technology has brought about the improvement of computer hardware, computing power, and large databases; thus, intelligent algorithms, such as machine learning, reinforcement learning and deep learning have gradually emerged, which have ushered in the great-leap-forward development of AI technology and produced several macro technical directions, such as computer vision, speech recognition, natural language recognition, decision planning, and big data analysis. Great breakthroughs have been made in the intelligent identification, understanding, and decision-making of data. During this period, many scholars published important works on AI (Table 1), and the important scientific discoveries in the development process of AI embodied important historical significance (Table 2).

In recent years, the rapid development of computer technology and the internet have granted AI intelligent algorithms for machine learning, reinforcement learning, and deep learning, in particular, convolution neural networks, cyclic neural networks, and recurrent neural networks. AI technology has ushered in development by great leaps and is, thus, being widely used in joint surgery.

Robot-assisted preoperative planning system includes mainly high-precision 3D reconstruction based on CT and MRI images, image-based determination of preoperative osteotomy area and osteotomy plane, joint prosthesis design and preparation, preoperative joint line measurement, biomechanical analysis of the knee joint after prosthesis implantation, and so on. Current robotic systems can be divided into three categories according to their working modes: active, semi-active, and passive. The commonly used robot systems are shown in Table 3.

The active robot does not require manual operation and operates according to the preoperative plan. However, the system has potential surgical risks, and its working process needs to be monitored by the doctor throughout to avoid accidents. The semi-active robot, also called the haptic robot, is used to perform surgical planning before the operation, and during the operation, the surgeon completes the operation with the help of the robotic arm. Moreover, if the intraoperative osteotomy exceeds the preoperative planning range, the system gives feedback to the surgeon by means of a sound and force feedback, and the robotic arm automatically stops the operation, thus ensuring that the surgical path and the range are consistent with those planned preoperatively [31,32].

**Figure 3:**
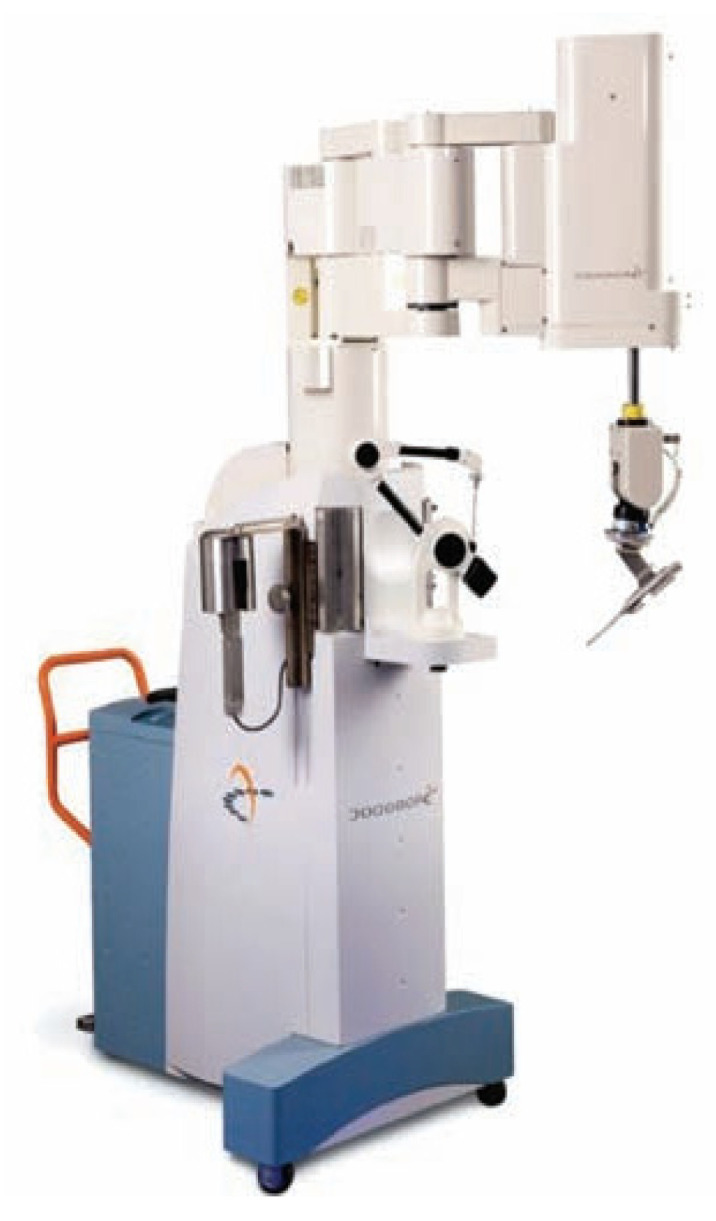
ROBODOC.

**Figure 4:**
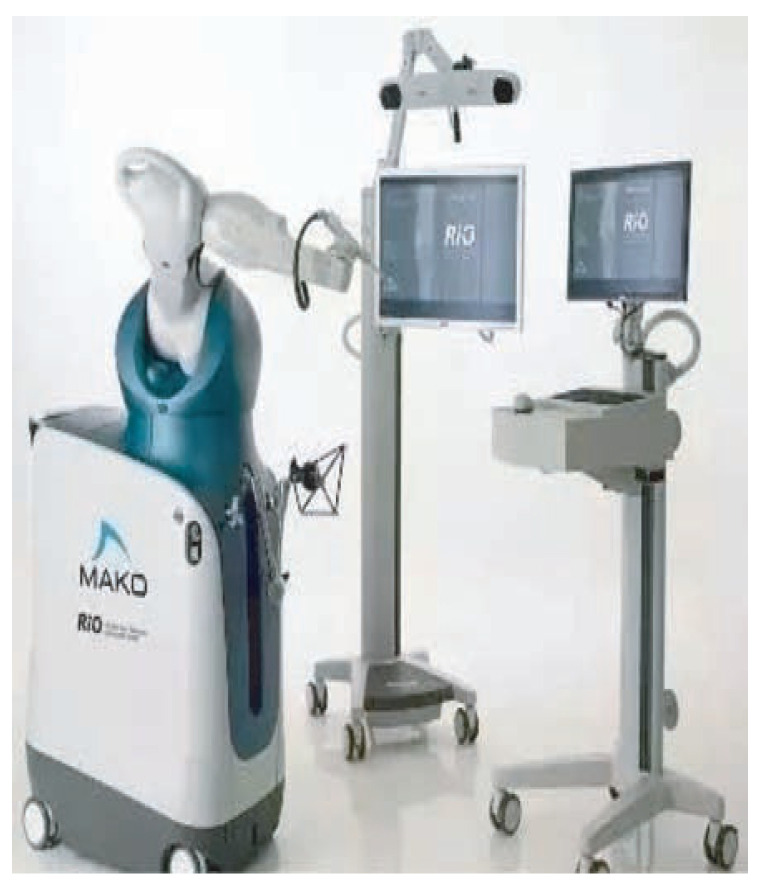
MAKO.

At present, several robotic-assisted arthroplasty surgery systems have been developed in the United States, Japan, Israel, and the United Kingdom, including the MARS surgical system developed by the Research Office of Mechanical Engineering Department in Israel [33], the 7-degree-of-freedom MIS-UKA system for robot-assisted orthopaedic surgery developed in collaboration with the University of Tokyo and Chiba University in Japan [34], and the ROBODOC (Figure 3) robot system developed by Integrated Surgical Systems Company in the United States [35]. Similarly, MAKOplasty is a semi-autonomous robotic system based on CT scanning and visual navigation developed by MAKO(Figure 4) [36], and ACROBOT is a semi-automatic TKA system developed by Imperial College Mechatronics in Medicine Laboratory, UK [37].

ROBODOC robots were first introduced for TKA in 2000, received FDA approval in 2008, and were renamed THINK robots in 2014. In addition to the ROBODOC and MAKO systems, there are also the NAVIO and iBlock systems. NAVIO is a handheld robot that can be manipulated by a surgeon [32]. The system was first approved by the FDA in 2012 for unicondylar replacement and patellar replacement and is now available for TKA. It does not require preoperative CT images and serves as an open robotic assist system for different prostheses from different manufacturers [32,38].

Currently, several hospitals in China are collaborating on the development of surgical robots for joint surgery. The computer-assisted orthopaedic surgery system, WATO, developed by the Department of Orthopaedics of Shanghai Changzheng Hospital and Shanghai Jiao Tong University Institute of Image Processing and Pattern Recognition in 2009, provides the surgeon with a surgical plan based on CT images before surgery, albeit with the disadvantage that the surgical incision is larger than that of conventional surgery [39]. The Gusheng Yuanhua Orthopaedic Surgery Robot, jointly developed by six hospitals, including the Department of Orthopaedics of the First Medical Centre of Chinese PLA General Hospital and Yuanhua Intelligent Technology (Shenzhen) Co., Ltd. (Heping Industrial Park, Heping Road, Yucui Community, Longhua Street, Longhua District, Shenzhen City, Guangdong Province, China) in 2020, also carries out preoperative planning on the basis of CT images when performing TKA. Notably, its human–machine interaction strategy is optimized with the habits of Chinese surgeons, and is, thus, more in line with the requirements of Chinese surgeons. Compared with the Mako robot, it is smaller in size and has a 7-degree-of-freedom manipulator with a larger range of motion and better flexibility. In addition, in 2021, the orthopaedic team of the Ninth People’s Hospital Affiliated with Shanghai Jiaotong University School of Medicine developed the Honghu orthopaedic surgical robot for TKA and reported five cases of TKA using the robot.

The significance of robot-assisted joint replacement is to greatly improve the accuracy of intraoperative operation, which benefits from three guarantees: (1) accurate measurement and planning of preoperative CT data; (2) the stable assistance of the mechanical arm during the operation; and (3) timely registration and monitoring during operation. The shortcomings and complications of early robotic joint surgery, after years of continuous correction and improvement, have no longer become the limiting factor of surgery.

It is generally accepted that excellent long-term postoperative results can be obtained when the postoperative knee varus or valgus is controlled within 3° after TKA surgery, but most of the current coronal lower extremity joint lines are controlled by relying on femoral intramedullary positioning and tibial extramedullary positioning. Further, the operation depends on the surgeon’s experience, which makes it difficult to achieve standardization and reproducibility of surgical outcomes. As can be seen from the above, the gradual deepening of the study of artificial intelligence has brought revolutionary changes in medicine, and concurrently, the preoperative planning of total knee arthroplasty in joint surgery is also moving towards the paradigm of precision medicine.

## 7. Conclusions

TKA requires accurate force line and soft tissue balance after operation. In order to achieve this goal, the planning of preoperative imaging of patients is particularly important. The application of computer-aided technology and artificial intelligence preoperative measurement can enable surgeons to control the osteotomy plane and angle more accurately. Although these advances have certain risks and disadvantages, in the field of joint surgery, AI assistance will still be the direction of future development and will also bring more breakthroughs.

## Figures and Tables

**Table 1:** Important AI publications.

Name	Author(s)
Artificial Intelligence: A Modern Approach	Stuart Russell, Peter Norvig
Human-Compatible Artificial Intelligence and the Problem of Control	Stuart Russell
The Sentient Machine: The Coming Age of Artificial Intelligence	Amir Husain
Deep Medicine–How Artificial Intelligence Can Make Healthcare Human Again	Eric Topol
life 3.0: Being Human in the Age of Artificial Intelligence	Max Tegmark
The Society of Mind	Marvin Minsky
DEEP MEDICINE	Eric Topol
Deep Learning	Ian Goodfellow, Yoshua Bengio, Aaron Courville

**Table 2:** AI scientific findings.

Date	Event
1956	Dartmouth Conference–birth of AI
1959	Arthur Lee Samuel put forward the concept of Advice Taker–birth of machine learning
concept	
1966	Joseph invented ELIZA, a robot that can chat–the first man–machine dialogue
1973	Waseda University in Japan has built the first humanoid robot, WABOT-1
1997	DEEP BLUE of IBM won the man–machine battle in the chess match
2006–2009	ImageNet was invented–the foundation of image recognition technology of AI
2016	The emergence of AlphaGo raised the world’s expectation of AI to an unprecedented level
2018–2019	IBM AI debate robot “IBM Project Debater” is the champion of human debate

**Table 3:** Robot systems (commonly used).

Robot Systems	Company	Classification	Application
ROBODOC	Think Surgical (Fremont, CA, USA)	active	Hip/knee arthroplasty
CASPAR	Universal Robot Systems Ortho (Odense, Denmark)	active	Hip arthroplasty
ACROBOT	Acrobot (London, UK)	active	Hip arthroplasty
Rio MAKO robot	Stryker (karamazu, MI, USA)	semi-active	Hip/knee arthroplasty
NAVIO	Blue Belt Technologues (Texas, USA)	semi-active	Condylar/knee arthroplasty
iBlock	OMNIlife (Fort, TX, USA)	semi-active	Knee arthroplasty
ROSA	Zimmer (Warsaw, IA, USA)	semi-active	Spinal surgery/knee arthroplasty

## Data Availability

Not applicable.
